# Identification of candidate genes and development of KASP markers for soybean pod-related traits using GWAS

**DOI:** 10.3389/fpls.2025.1680918

**Published:** 2025-11-26

**Authors:** Zicong Liang, Nianhua Qi, Ruoning Li, Ruijia Gao, Ruichao Guo, Jiayi Li, Yutong Han, Nan Xie, Wei Zhao, Xingdong Yao, Futi Xie

**Affiliations:** 1 College of Agriculture, Shenyang Agricultural University, Shenyang, China; 2 College of Agriculture, Agriculture School of Liaodong University, Dandong, China

**Keywords:** soybean, GWAS, yield, KASP marker, pod-related traits

## Abstract

Soybean (*Glycine max* [L.] Merr.) is a crop characterized by rich content of oil and protein in seeds, enhancing both yield and quality is considered a pressing challenge in current soybean research and production. Soybean yield is determined by individual traits, including seed number per plant, seed weight per plant, pod number per plant, pod weight per plant and 100−seed weight. Here, 338 resequenced soybean varieties (or lines) were evaluated under two planting densities for five pod−related traits. Substantial variation was detected among the 338 accessions under both densities, and all phenotypic traits followed a normal distribution. A total of 47 and 56 significant SNPs were identified respectively under high and low planting densities through genome−wide association studies (GWAS). Among them, eight SNPs were repeatedly detected across at least two planting densities or environments, and were significantly associated with the seed number per plant (SNPP), seed weight per plant (SWPP) and 100−seed weight (HSW). Based on linkage disequilibrium (LD) analysis, haplotype analysis, gene functional annotation, and qRT−PCR validation, *Glyma.20G116200* and *Glyma.13G162800* were identified as key genes associated with HSW and SNPP, respectively. Based on this, a KASP marker, S20_35808042 (G/C), was developed and successfully validated in 97 soybean accessions. In summary, these findings hold substantial value for soybean improvement, providing new insights into the genetic architecture of pod−related traits and establishing a conceptual foundation for marker−based selection in breeding programs.

## 1 Introduction

Soybean (*Glycine max* [L.] Merr.) is regarded as one of the most important economic and oilseed crops and is widely cultivated worldwide due to its rich seed content of oil and protein (Hina et al., 2022) (https://soystats.com, accessed on 1 July 2025), Statistics show that the global average yield increase of soybean is slowing down and remains much lower than maize and wheat (Bhat et al., 2025). Therefore, improving soybean yield and quality remains a critical challenge in current crop production and breeding efforts. Seed yield and its component traits in soybean represent complex quantitative characteristics governed by multiple genes and strongly affected by environmental factor (Qi et al., 2014). Soybean yield is determined by several individual traits, including seed number per plant, seed weight per plant, pod number per plant, pod weight per plant and 100−seed weight (Wang et al., 2024). Soybean breeders aim to maximize the use and improvement of these traits to enhance overall yield. Planting density is also recognized as a critical agronomic variable that significantly affects dry matter accumulation and yield in field crops (Friedman et al., 2024). Increasing planting density can improve the efficiency of canopy light interception; however, excessive density intensifies inter−plant competition, leading to lodging and reduced per−plant yield, ultimately limiting total productivity. Therefore, identifying an optimal balance between planting density and pod−related traits in soybean is essential for enhancing productivity and remains a pressing issue in current agricultural studies (Zhang et al., 2015).

A considerable number of investigations involving the construction of genetic linkage maps have dissected the genetic architecture of pod−related traits, aiming to identify quantitative trait loci (QTLs) associated with these traits using both bi−parental and multi−parental populations in soybean (Su et al., 2019; Luo et al., 2022; Han et al., 2012). Previous studies have demonstrated that, under varying planting densities, the number of pods and seeds per plant, seed size, and seed weight are significantly associated with soybean yield (Bianchi et al., 2020; Kitabatake et al., 2020). The coordinated regulation of these traits is regarded as a key physiological basis for yield determination. To date, SoyBase has recorded over 500 quantitative trait loci (QTLs) that are linked to pod-relates traits in soybean, and they are distributed across all 20 soybean chromosomes. In a previous study, a population of 207 F_2_:_3_ progenies developed from a cross between Brazilian cultivar CS3035P−TA276−1−5−2 and UFVS2012 was used for QTL mapping of yield−related traits (Rodrigues et al., 2016). A total of 30 QTLs significantly associated with pod-related traits were identified using composite interval mapping, and 4 QTLs displaying additive effects were subsequently utilized in marker−assisted selection (MAS) programs. Another study reported that a recombinant inbred line (RIL) population developed from the hybridization of PI595843 (PI) and WH was utilized, leading to the identification of 38 QTLs significantly linked to 100-seed weight, including four major−effect QTLs (Xu et al., 2023). Golestan Hashemi et al. (2018) employed a RIL population comprising 147 lines derived from the hybridization of the U.S. cultivar Charleston and the Chinese cultivar Dongnong 594, was employed to detect two QTLs with significant correlations to seed weight per plant. In addition, four candidate genes were identified on chromosome 1. However, these genes responsible for these QTLs, as well as the biological pathways through which they influence yield-related characteristics, are still not well understood. With the advancement of genomic analysis technologies, genome−wide association studies enable precise localization of causal genes by leveraging extensive natural variation and high−density SNP markers. Zhang et al. conducted a GWAS using a soybean panel of 1,714 accessions and identified 35 stable association signals significantly related to 100−seed weight (HSW). In addition, a stable QTL hotspot, qSW17.1, was detected on chromosome 17 (Zhang et al., 2024). In another previous study, researchers identified four novel QTLs significantly associated with four yield−related traits, based on an association panel comprising 211 soybean varieties. In addition, several haplotype alleles were revealed to contribute to the phenotypic variation of these traits (Bhat et al., 2022).

In soybean, multiple pivotal genes associated with pod-related traits, such as *GmKIX8−1*, *PP2C−1*, and *SW16.1*, have been successfully cloned (Nguyen et al., 2021; Lu et al., 2017; Chen et al., 2023). Nguyen et al. (2021) identified *GmKIX8−1* as a major−effect gene underlying a key QTL for HSW, using a forward genetics approach combined with CRISPR/Cas9 gene editing technology. Up to now, genome-wide association analyses have facilitated the discovery and functional validation of multiple genes involved in soybean yield-related traits, including *GmSWEET10a*, *GmST05*, and *GmGA30ox* (Li et al., 2024; Duan et al., 2022; Hu et al., 2022). For instance, *GmST05* enhances seed size and contributes to yield improvement by influencing the expression level of *GmSWEET10a*, which in turn alters the oil and protein composition of seeds (Duan et al., 2022). These genes participate in multiple signaling pathways, such as plant hormone signaling and transcriptional regulation, which help elucidate the molecular mechanisms underlying yield formation. However, the genetic mechanisms of the interactions among pod−related traits remain poorly understood.

Therefore, this research was conducted to clarify the genetic architecture underlying pod−related traits in soybean, to explore the genetic variation among these traits, and to identify genes and molecular markers that facilitate genetic enhancement of yield-related characteristics in soybean. A natural population of 338 soybean accessions originating from similar latitudes were used to minimize the environmental effects caused by latitude−related variation in growth and development. The population was planted at a single location with consistent latitude under two planting density conditions (150,000 and 300,000 plants/ha) to simulate ecological environments under current high−density cultivation trends. were performed for five pod-related traits within this population. We integrated genome-wide association mapping with haplotype analysis and qRT–PCR analysis to identify candidate loci and genes, and developed KASP molecular markers accordingly. These results provide important implications for advancing molecular breeding strategies and evolutionary research of high−yield soybean cultivars. They facilitate the discovery of molecular markers linked to pods−related traits and promote practical application of breeding strategies for yield improvement in soybean.

## 2 Materials and methods

### 2.1 Plant material and phenotypic analysis

To minimize the potential impacts of cross−latitude germplasm introduction on soybean growth, development, and lodging resistance, a natural population consisting of 338 soybean germplasm resources was established (**Supplementary Table S4**). This population primarily included varieties (lines) bred in different eras in Liaoning Province, supplemented with partial varieties introduced from Japan and the United States at similar latitudes. Two planting densities were set: low and high (150,000 and 300,000 plants/ha), which were used in the materials and methods section of the research. In 2022 and 2023, all soybean accessions were cultivated at three experimental sites affiliated with Shenyang Agricultural University, located in Liaoning Province, Shenyang (E1_HD/LD: SH 2022; E2_HD/LD: SH 2023, N41°82’,E 123°57’), Hunnan District (E3_HD/LD: 2023HN, N 41°75’,E 123°69’), and Pulandian District, Dalian (E4_HD/LD:2023DL,N 39°54’,E 122°20’). Manual thinning was performed at the V2 growth stage to ensure appropriate plant density. The soil type at all three locations was loam. All field trials were managed according to standard agronomic practices, with consistent soil types and nutrient management strategies implemented across the three experimental sites.

At the maturity stage (R8), four individual soybean plants were selected randomly from each plot. Five pod−related traits were measured: PNPP (pod number per plant): The number of valid pods per plant was determined by manual counting. SNPP (seed number per plant): The number of well-developed seeds per plant was determined by manual counting after threshing. HSW (100−seed weight): A random sample of 100 seeds from the bulk harvest of each genotype was weighed. PWPP (pod weight per plant) and SWPP (seed weight per plant): Pod dry weight per plant (before threshing) and seed dry weight per plant (after threshing).Each soybean accession was evaluated using four biological replicates in the experimental setup.

### 2.2 Whole−genome re−sequencing and SNP calling

In 2022, young leaves from 338 soybean genotypes were flash-frozen with liquid nitrogen and pulverized to break cell structures, after which genomic DNA was isolated from the resulting homogenate employing the CTAB extraction technique (Carey et al., 2023). DNA libraries were prepared with the MGIEasy Universal DNA Library Prep Kit V1.0 (Product No.: 1000005250; MGI, Shenzhen, China) and sequenced on the BGISEQ–T7 platform (Liu et al., 2020; Zhou et al., 2019)). Raw reads were filtered to remove those containing >10% ambiguous bases (N) or a Phred quality score <10. High−quality reads were mapped to the Glycine max Wm82.a2.v1 reference genome using BWA (v0.7.17) (Li and Durbin, 2010). Variant calling was performed using BCFtools (v1.12), SNPs with a missing data rate below 15% and a minor allele frequency (MAF) greater than 0.05 were retained, and genotype imputation was subsequently performed using Beagle (v5.1), yielding a final dataset of 4,432,394 SNPs for downstream analyses.

### 2.3 Genome−wide association study

The 4,432,394 high-quality SNPs were analyzed against the Best Linear Unbiased Predictors (BLUPs) computed from the multi-environment phenotypic data using the Efficient Mixed-Model Association eXpedited (EMMAX) software (version: 20120205) to detect significant marker-trait associations. BLUPs were estimated using a mixed model with genotype as a random effect, while accounting for the fixed effects of environment and replication block. GWAS were performed using a mixed linear model (MLM) incorporating the population structure matrix (Q) and kinship matrix (K), with all other parameters set to default values. “Principal component analysis (PCA) was performed using PLINK (v1.9) to calculate the first 20 principal components for the experimental population. Significance testing for each principal component was conducted using the twtable algorithm implemented in EIGENSOFT(v7.2.1). The top two principal components showing significant population stratification (P < 0.05) were selected as covariates for downstream analyses. A kinship matrix was generated using EMMAX (v20120205) based on genome-wide SNP data, with genetic relatedness between samples estimated using the Balding-Nichols model. The mean values of the four single plants for each trait in soybean accession were used for the association mapping for this experiment. A significance threshold of −log_10_(*P*) = 5.0 was set, and the results were graphically represented using Manhattan and Q–Q plots.

### 2.4 Screening and prediction of candidate genes and haplotype analysis

Haplotype identification was performed through an integrated approach combining linkage disequilibrium (LD) decay analysis with sliding−window scanning. Fixed physical−distance sliding windows were implemented with 100−kb window size and 50−kb step length. The selected window size was informed by the average linkage disequilibrium (LD) decay distance of 50 kb estimated among the 338 soybean genotypes (Liang et al., 2025). Single nucleotide polymorphisms (SNPs) showing significant associations within a 100 kb chromosomal span were clustered into a single physical region for further analysis. The single nucleotide polymorphism (SNP) exhibiting the maximum −log_10_(*P*) value was designed as the peak locus, and a 100 kb window (50 kb upstream and 50 kb downstream) around this peak was defined as the confidence interval. Haplotype block partitioning was performed using LDBlockShow software (v1.4), analyzing all SNPs within ± 50 kb regions centered on peak SNP positions. All SNPs located between the 5’ and 3’ untranslated regions (UTRs) of genes within the candidate intervals are retrieved from the resequencing dataset. For haplotype analysis, SNPs within the coding sequence (CDS) region of the target gene are extracted using Excel software, based on the same VCF file employed in the GWAS analysis.

### 2.5 qRT−PCR analysis

Two soybean cultivars were selected as experimental materials: Liaodou 11 (LD 11), which has a relatively large 100−seed weight (average HSW of 22.02 g over two years), and Shennong 12 (SN 12), which has a relatively small 100−seed weight (average HSW of 15.80 g over two years). Fresh seeds were collected at the R6 stage. Total RNA was extracted employing the RNAprep Pure Plant Plus Kit (Tiangen Biotech, Beijing, China), followed by reverse transcription and quantitative PCR (qRT-PCR) analysis. All primer sequences (**Supplementary Table S5**) were designed using NCBI’s Primer-BLAST tool (https://www.ncbi.nlm.nih.gov/tools/primer-blast/). The internal reference gene Actin was utilized as the internal control, and relative expression levels were calculated using the 2^−ΔΔCt^ method. Each biological sample was analyzed in triplicate with three independent replicates.

### 2.6 Development of KASP marker

Three allele−specific primers (F1, F2, and R) were designed for the KASP marker site S20_35808042. The KASP−PCR primers were designed using Primer3 software, with melting temperatures (Tm) ranging from 55°C to 65°C (**Supplementary Table S6**). Marker validation and sample detection were carried out using the HCSCI (HanchenGuangyi) system. PCR amplification was performed in a Matrix Cycler high−throughput thermocycler, and fluorescence signals were detected with the Matrix Scanner. SNP genotyping was conducted on the HCSCI Master platform based on the criteria of clear genotype clustering and the absence of specific amplification in the no−template control (NTC).

### 2.7 Data analysis

Statistical analyses were conducted using IBM SPSS Statistics 26.0 (SPSS, Inc., Chi-cago, IL, USA) and R software. For each agronomic characteristic, the mean, standard deviation (SD), and coefficient of variation (CV) were calculated using Microsoft Excel (Microsoft Corporation, Redmond, WA, USA). Best linear unbiased predictions (BLUPs) for each trait were estimated using the lme4 package (v1.1−35) in R (Bates et al., 2015). For multi−environment data, observed phenotypic values were used directly, with location, year, and individual sample treated as random effects in the model. Correlation analysis, Manhattan plots, and Q–Q plots were visualized using the R packages GGally v2.2.1 and CMplot v3.8.1, respectively. Expression profiles of soybean genes across different tissues were retrieved from the Phytozome database, and heatmaps of candidate genes in target regions were generated using GraphPad Prism (v10.0.1). Phenotypic variation across haplotypes was analyzed by one−way ANOVA in IBM SPSS Statistics 27, with results visualized using box plots in GraphPad Prism (v10.0.1).

## 3 Results

### 3.1 Phenotypic variation of soybean pods−related traits under different planting densities

**Figure 1:**
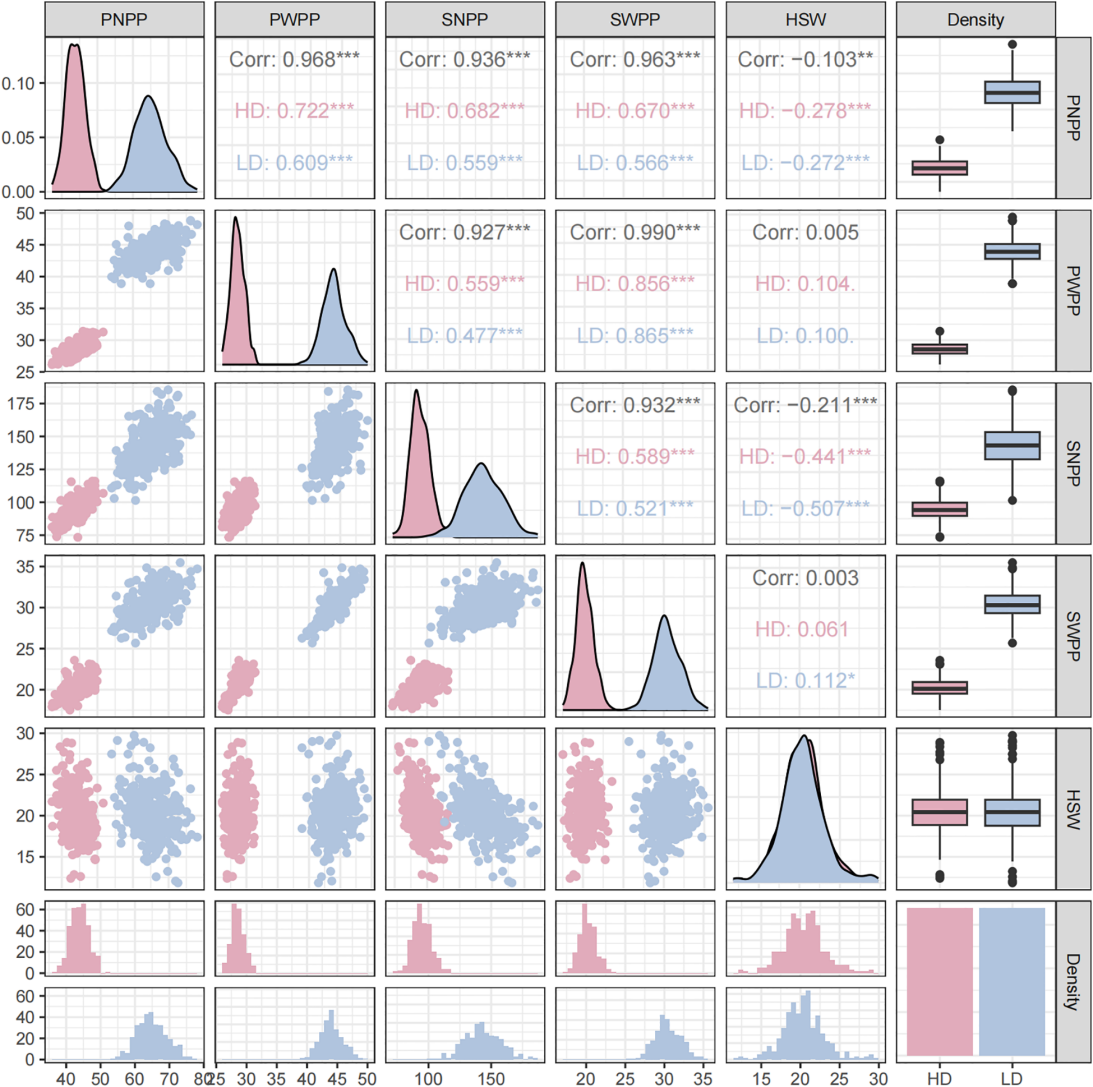
Correlation analyses, frequency distribution plots, and overall boxplots of pod−related traits under two planting densities based on BLUP values. (Blue: 300,000 plants/ha; Pink: 150,000 plants/ha). PNPP (pod number per plant), SNPP (seed number per plant), PWPP (pod weight per plant), SWPP (seed weight per plant), HSW (100−seed weight). **indicates a significant correlation (p < 0.01), *** indicates a significant correlation (p < 0.001).

Pod−related phenotypes of 338 soybean accessions were evaluated across 3 environments over 2 years. Descriptive statistics and frequency distributions are presented in **Supplementary Table S1**. Based on BLUP values, under high planting density (300,000 plants/ha), the ranges of PNPP, SNPP, PWPP, SWPP, and HSW were found to be 37.25–51.63, 73.42–116.08, 26.17–31.39 g, 17.52–23.58 g, and 12.39–28.90 g, respectively.CV value for these phenotypes ranged from 4.11 to 12.62%. Under low planting density (150,000 plants/ha), the ranges of PNPP, SNPP, PWPP, SWPP, and HSW were observed to range sequentially from 54.01–78.06, 101.42–185.35, 38.88–49.36 g, 25.68–35.48 g, and 11.84–29.75 g, respectively. CV value ranged from 4.20 to 13.36% (**Table 1**). In comparison with high density, the ranges of phenotypic variation were larger under low density. The observed broad-sense heritability (H²) for the five traits was moderate to high (33.0–88.9%), confirming that their variation was significantly controlled by genotypic as well as environmental factors. These traits exhibited normal or skewed distributions under different densities (**Supplementary Table S1**; **Figure 1**). These results demonstrated that pod−related traits in soybean displayed extensive phenotypic variation.

**Table 1:** Statistical and differential analysis of multi-environment BLUPs for pod-related traits in soybean.

Trait	Density	Range	Mean ± SD	CV(%)	Skewness	Kurtosis	H^2^(%)
PNPP	High density	37.25 − 51.63	43.86 ± 2.62	6.06	−0.28	0.13	40.4
	Low density	54.01 − 78.06	64.94 ± 4.46	6.87	−0.20	0.21	41.4
SNPP	High density	73.42 − 116.08	94.62 ± 7.43	7.85	0.17	0.28	66.0
	Low density	101.42 − 185.35	143.29 ± 14.67	10.23	0.03	0.10	68.4
PWPP/g	High density	26.17 − 31.39	28.61 ± 1.08	4.11	−0.31	0.17	35.7
	Low density	38.88 − 49.36	44.00 ± 1.85	4.20	0.02	0.18	33.0
SWPP/g	High density	17.52 − 23.58	20.22 ± 1.06	5.25	0.01	0.25	33.0
	Low density	25.68 − 35.48	30.41 ± 1.67	5.48	−0.02	0.20	37.3
HSW/g	High density	12.39 – 28.90	20.48 ± 2.59	12.62	0.91	0.21	88.9
	Low density	11.84 − 29.75	20.40 ± 2.73	13.36	1.43	0.33	88.9

Pearson correlation analyses of the five pod−related traits revealed that highly significant positive correlations were observed among PNPP, SNPP, PWPP, and SWPP under both planting densities, with correlation coefficients spanning from 0.477 to 0.856. In contrast, HSW exhibited highly significant negative correlations only with PNPP and SNPP, with correlation coefficients ranging from –0.272 to –0.507. Notably, HSW showed a significant positive correlation with SWPP only under low density (**Figure 1**). These results demonstrated that planting density could significantly affect the variation trends of yield traits and indicated their potential utility in GWAS analyses.

### 3.2 Results of genome−wide association studies

**Figure 2:**
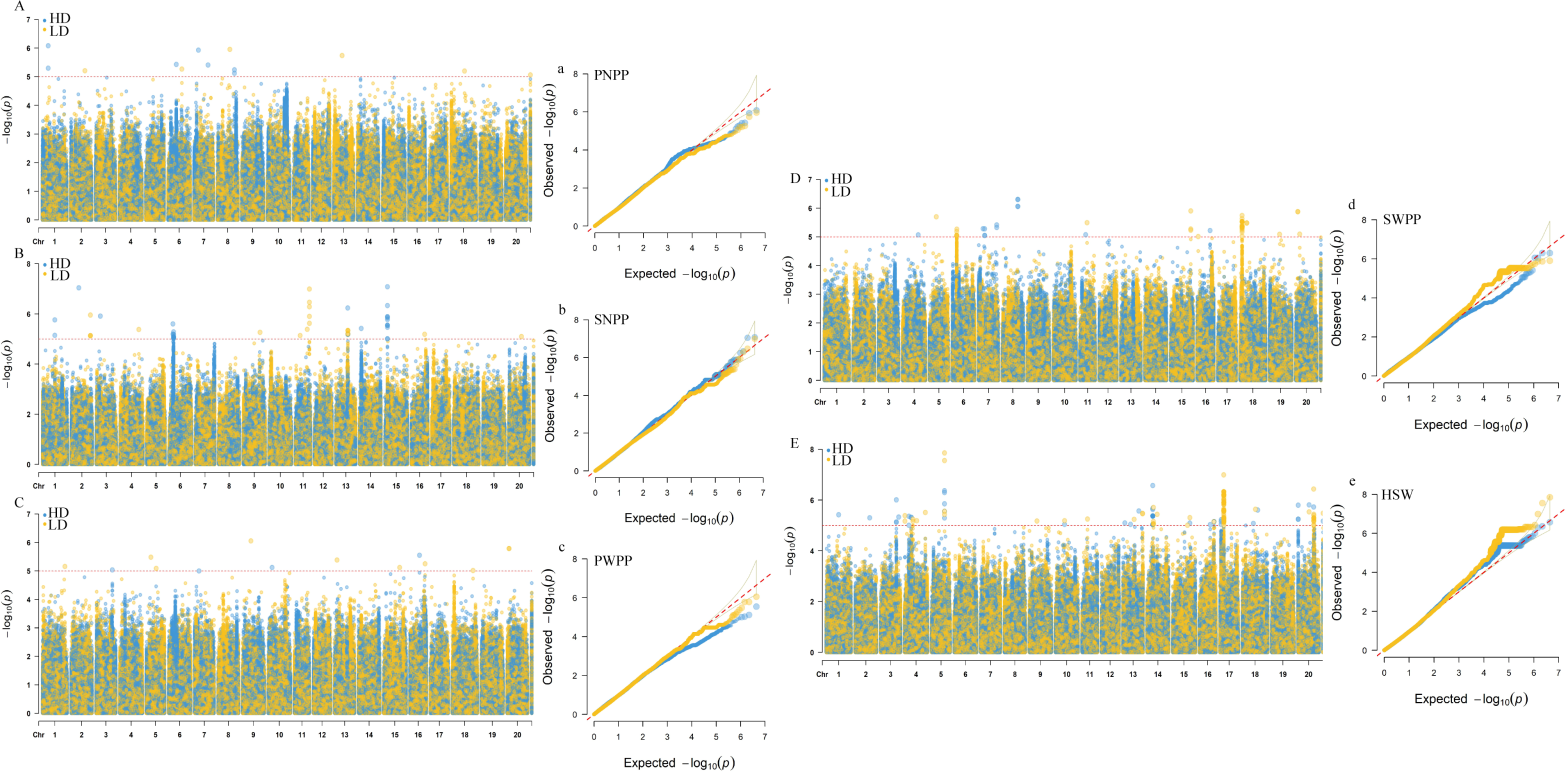
GWAS results for pod−related traits under two planting densities based on BLUP values. **(A−E)** Manhattan plots for PNPP (pod number per plant), SNPP (seed number per plant), PWPP (pod weight per plant), SWPP (seed weight per plant) and HSW (100−seed weight). **(a−e)** Q−Q plots for PNPP, SNPP, PWPP, SWPP, and HSW. HD, 300,000 plants/ha; LD, 150,000 plants/ha.

Based on BLUP values, GWAS analyses for soybean pod−related traits under two planting densities were performed using the MLM model, with correction for (K) and PCA results. Under high planting density, 46 SNPs distributed across 17 chromosomes were found to be associated with yield traits, individually explaining 5.64-8.25% of the phenotypic variance. Among these findings, 21 SNPs were associated with HSW, 9 with SNPP, 7 with SWPP, 5 with PNPP, and 4 with PWPP. Under low planting density, 52 SNP loci linked to pod-related characteristics were identified across 18 chromosomes, with each locus explaining 5.69-8.14% of the phenotypic variation. Among these,19 with HSW, 9 with SNPP, 10 with SWPP, 5 with PNPP, and 9 with PWPP (**Supplementary Table S2**). In this study, SNPs that were detected across different planting densities or environments, either replicated or closely linked, were identified as important pod−related SNPs. SNPs detected under a single condition were excluded from candidate gene analyses. These findings indicated that 8 significant SNP loci were consistently identified in at least two planting densities or environmental conditions. Among these, one locus stably and significantly associated with HSW was detected on each of chromosomes 5, 14, 15, 17, and 20 (**Table 2**; **Figure 2**); one locus significantly associated with SNPP was detected on chromosome 13; and one locus each on chromosomes 7 and 19 was detected to be significantly associated with SWPP.

**Table 2:** Highly significant SNP markers associated with pods−related traits consistently detected under both planting densities.

Traits	Environments	Significant SNP	Chr.	Position	*P−value*	MAF	PVE(%)
HSW	Blup_HD and Blup_LD	Chr05.:29526655	5	295,266,55	6.36−7.85	0.06	7.38−9.18
	Blup_HD and Blup_LD	Chr15.:39442814	15	394,428,14	5.09−5.31	0.12	5.79−6.05
	Blup_HD and Blup_LD	Chr20.:35828042	20	358,280,42	5.72−6.43	0.07	6.85−7.43
	Blup_HD and E4_HD	Chr14.:12805909	14	128,059,09	5.29−6.57	0.06	6.67
	Blup_HD and E1_HD	Chr17.:2491941	17	2,491,941	5.32−5.97	0.13	5.96−9.61
SNPP	Blup_HD and Blup_LD	Chr13.:27800164	13	278,001,64	5.21−5.33	0.06	5.94−6.09
SWPP	Blup_HD and E2_HD	Chr07.:9833871	7	9,833,871	5.16−5.28	0.44	5.93−6.02
	Blup_LD and E2_LD	Chr19.:23028572	19	230,285,72	5.10−5.09	0.06	5.93−7.78

HD, High density; LD, Low density; Blup_HD, Environment-specific BLUPs under high planting density; Blup_LD, Environment-specific BLUPs under low planting density. E1_HD, SH2022 under high planting density; E2_HD, SH2023 under high planting density; E4_HD, 2023DL under high planting density.

**Figure 3:**
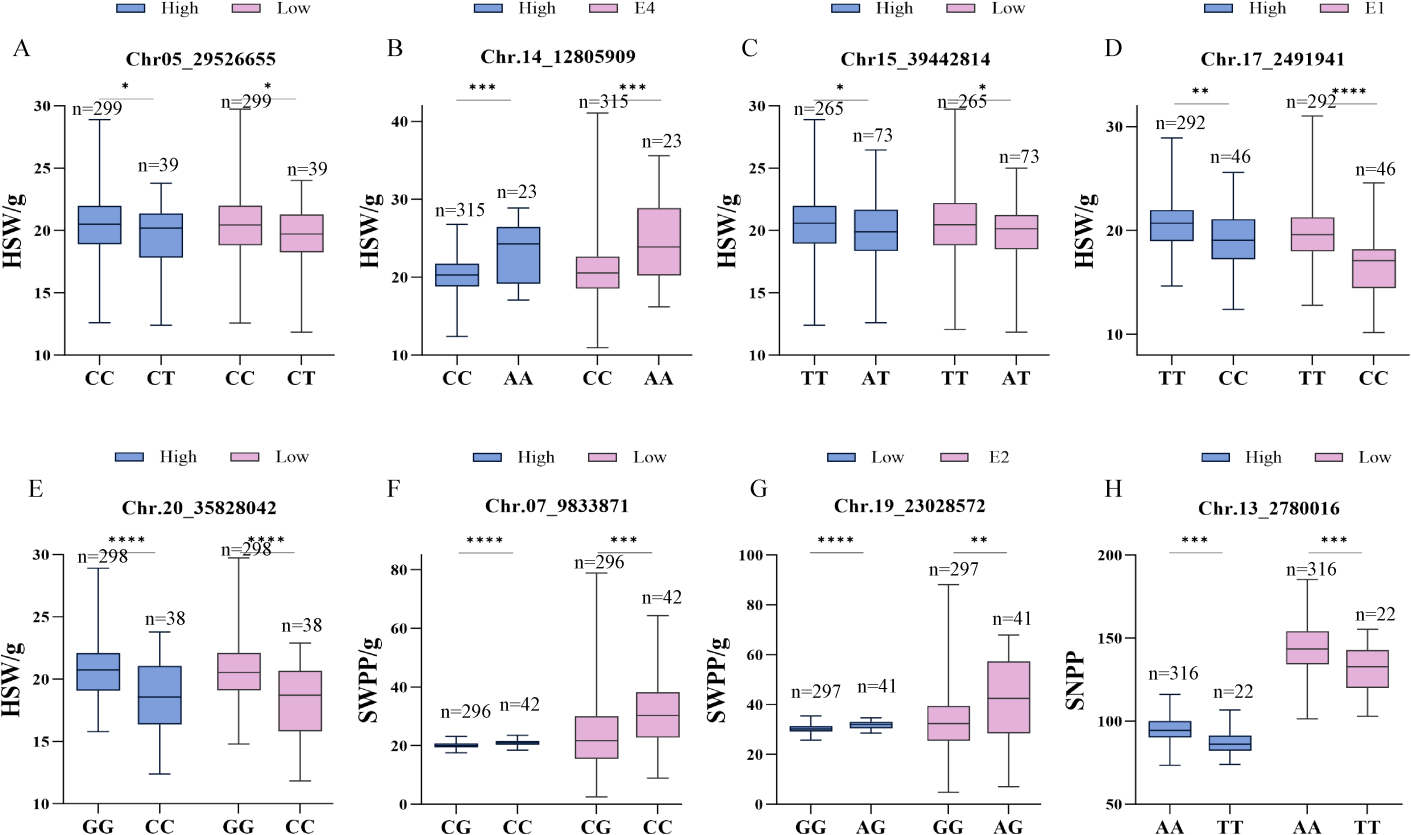
Phenotypic distributions of soybean germplasm resources across alleles of highly significant single nucleotide polymorphism (SNP) markers. **(A−H)** SNP05_29526655, SNP14_12805909, SNP15_40561307, SNP17_2491941, SNP20_35828042, SNP07_9833871, SNP19_23028572, SNP13_2780016. (*,**,*** and **** statistically significant phenotypic differences among haplotypes as determined by one-way ANOVA, respectively (P < 0.05,P < 0.01, P < 0.001,P < 0.0001).

Haplotype analysis was performed on the significantly identified loci to determine allelic combinations of multiple co−inherited loci, and subsequently to analyze their association with soybean pods−related traits. **Figure 3** illustrates the phenotypic distributions corresponding to the major alleles of these highly consistent and important SNPs. Pods−related phenotypes exhibited the same trend and showed significant differences among different alleles across various planting densities or environments. Among the 5 SNP loci associated with HSW, one important SNP locus was identified on each of chromosomes 5, 15, 17, and 20; germplasm carrying the minor alleles at these loci exhibited smaller HSW. In contrast, germplasms carrying the major allele of the SNP on chromosome 14 displayed smaller HSW (**Figures 3A−E**). For SWPP, the minor alleles at SNP07_9833871 (Chr. 7) and SNP19_25028572 (Chr. 19) were associated with higher seed weight per plant (**Figures 3F−G**). For SNPP, germplasms carrying the major allele of SNP13_2780016 (Chr. 13) exhibited the higher SNPP, with average counts of 132.21 and 97.09 under high and low densities, respectively (**Figure 3H**).

### 3.3 Identification of candidate genes

To identify the causal genes underlying variations associated with soybean yield, we predicted genes located within a 100 kb genomic region surrounding significant SNPs, based on linkage disequilibrium patterns derived from the above results. Functional annotation of soybean genes was performed using orthologous genes in Arabidopsis to determine the pathways involved in the determination of these traits. Potential genes were identified by integrating soybean gene IDs and protein annotation information retrieved from SoyBase to predict the biological functions performed by the genes. Among the 5 SNPs significantly associated with HSW, *Glyma.14G111800*, *Glyma.14G112000*, *Glyma.15G219900*, and *Glyma.20G116200* were identified as candidate genes. Their functions mainly include encoding aspartate aminotransferase, C2H2 and C2HC zinc finger superfamily proteins, etc. (**Supplementary Table S3**). Among the 2 SNPs significantly associated with SWPP, *Glyma.07G102600* and *Glyma.07G102700* were recognized as possible candidate genes. Their functions mainly include being core components of the C/D box snoRNP (small nucleolar ribonucleoprotein) complex, involved in rRNA processing and ribosome biogenesis, and encoding N−myristoyltransferase genes. Among the 1 SNP significantly associated with SNPP, *Glyma.13G162800* and *Glyma.13G163000* were identified as candidate genes, which mainly include S−adenosyl−L−methionine−dependent methyltrans-ferases superfamily protein and Monogalactosyldiacylglycerol Synthase 2 (a member of the glycolipid synthase family).

### 3.4 Haplotype analysis of candidate genes

**Figure 4:**
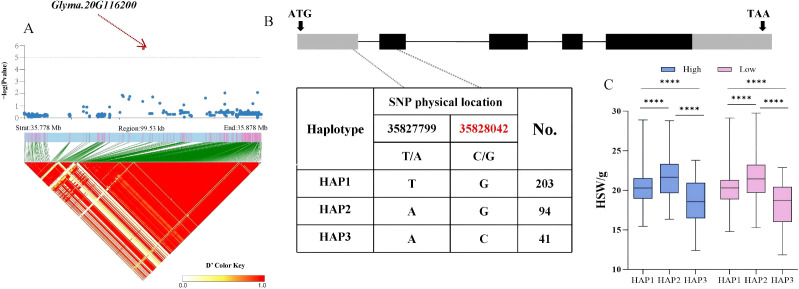
Analysis of the association region and haplotype of *Glyma.20G116200*. **(A)** Regional Manhattan Plot and Linkage Disequilibrium (LD) Heat Map of the Region of *Glyma.20G116200*. **(B)** Gene Structure and Variation of *Glyma.20G116200*. **(C)** Phenotypic Differences of Different Haplo-types of *Glyma.20G116200* for HSW. (**** respectively indicate significant phenotypic differences between different haplotypes, *p<0.0001*).

**Figure 5:**
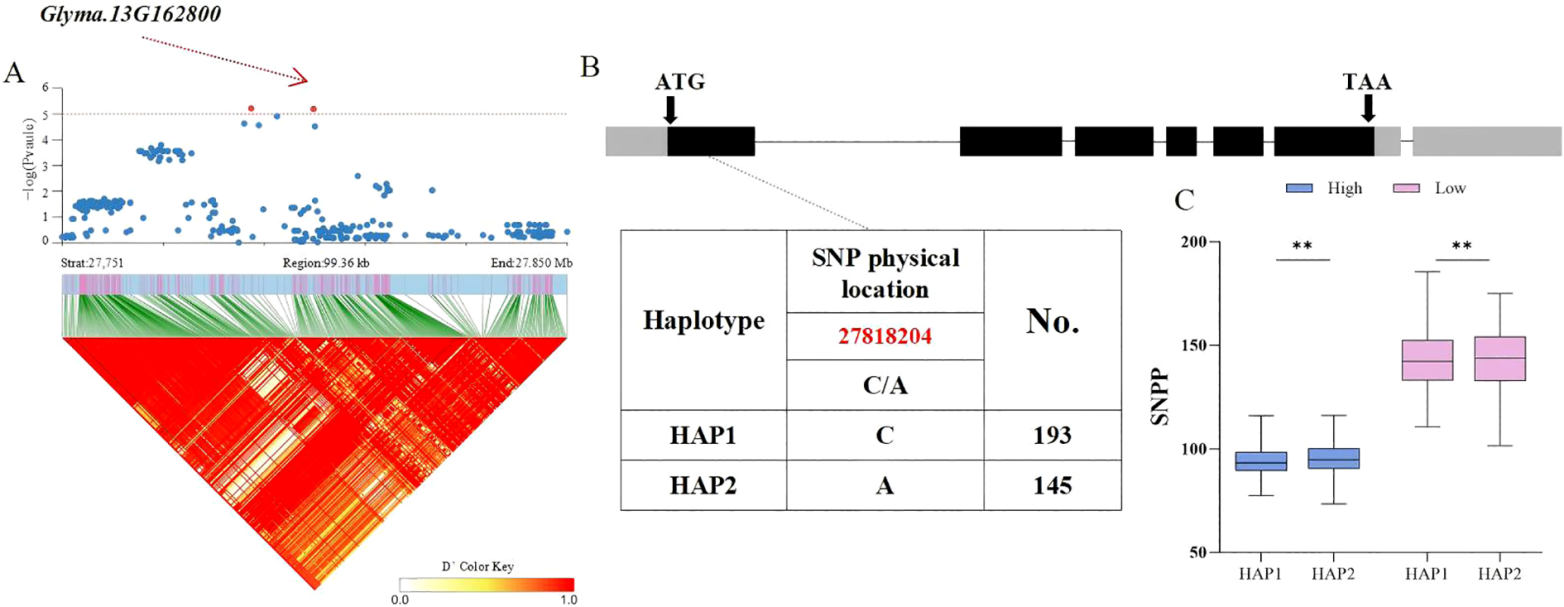
Analysis of the Association Region and Haplotype of *Glyma.13G162800*. **(A)** Regional Manhattan Plot and Linkage Disequilibrium (LD) Heat Map of the Region of *Glyma.13G162800*. **(B)** Gene Structure and Variation of *Glyma.13G162800*. **(C)** Phenotypic Differences of Different Haplo-types of *Glyma.13G162800* for SNPP. (** respectively indicate significant phenotypic differences between different haplotypes, *p<0.001*).

Based on the above findings, whether candidate genes exhibited polymorphism was determined by identifying phenotypic differences among haplotypes within the soy-bean population. Therefore, the distribution of significant non−synonymous SNPs in the candidate genes from the database was investigated. For the HSW trait, the *Glyma.20G116200* gene, located in the LD block of SNP Chr20:35828042, encodes a C2H2 and C2HC zinc finger superfamily protein. A total of 338 soybean accessions were divided into 3 haplotypes (HAP1 = 203, HAP2 = 94, HAP3 = 41) by two SNPs with variations, with a C/G non−synonymous mutation occurring at 35,828,042 bp in the coding region. Statistical tests revealed that significant differences in HSW among the three haplotypes were ob-served under both planting densities, with HAP3 (AC) exhibiting a smaller HSW. The average HSW of HAP3 under high and low planting densities was 18.32 g and 18.12 g, respectively *(p = 10.2 × 10^−4^, p = 6.1 × 10^−5^, p = 4.2 × 10^−8^*, *p = 3.8 × 10^−5^, p = 8.1 × 10^−5^, p = 1.2 × 10^−8^*) (**Figure 4**). For the seed number per plant (SNPP) trait significantly associated with SNP Chr13:27800164, the *Glyma.13G162800* gene in the LD block encodes an S−adenosyl−L−methionine−dependent methyltransferase superfamily protein. A total of 338 soybean accessions were divided into 2 haplotypes (HAP1 = 193, HAP2 = 145) by one SNP with variation, with a C/A non−synonymous mutation occurring at 278,182,04bp. Statistical tests revealed that highly significant differences in SNPP between the two haplotypes were observed under both planting densities, with HAP2 (A) exhibiting more seeds per plant (**Figure 5**). The average SNPP of HAP2 under high and low planting densities were 90.35 and 139.25, respectively, which increased by 1.03% and 0.4% compared to HAP1 (*p = 0.002, p = 0.005*). In recent years, breeders have preferred to develop density−tolerant soybean varieties, characterized by smaller HSW and more seeds per plant; thus, HAP3 of *Glyma.20G116200* and HAP2 of *Glyma.13G162800* were identified as elite haplotypes. Drawing from the findings above, *Glyma.20G116200* and *Glyma.13G162800* were recognized as pod-related candidate genes in soybean.

### 3.5 qRT−PCR validation of candidate genes

**Figure 6:**
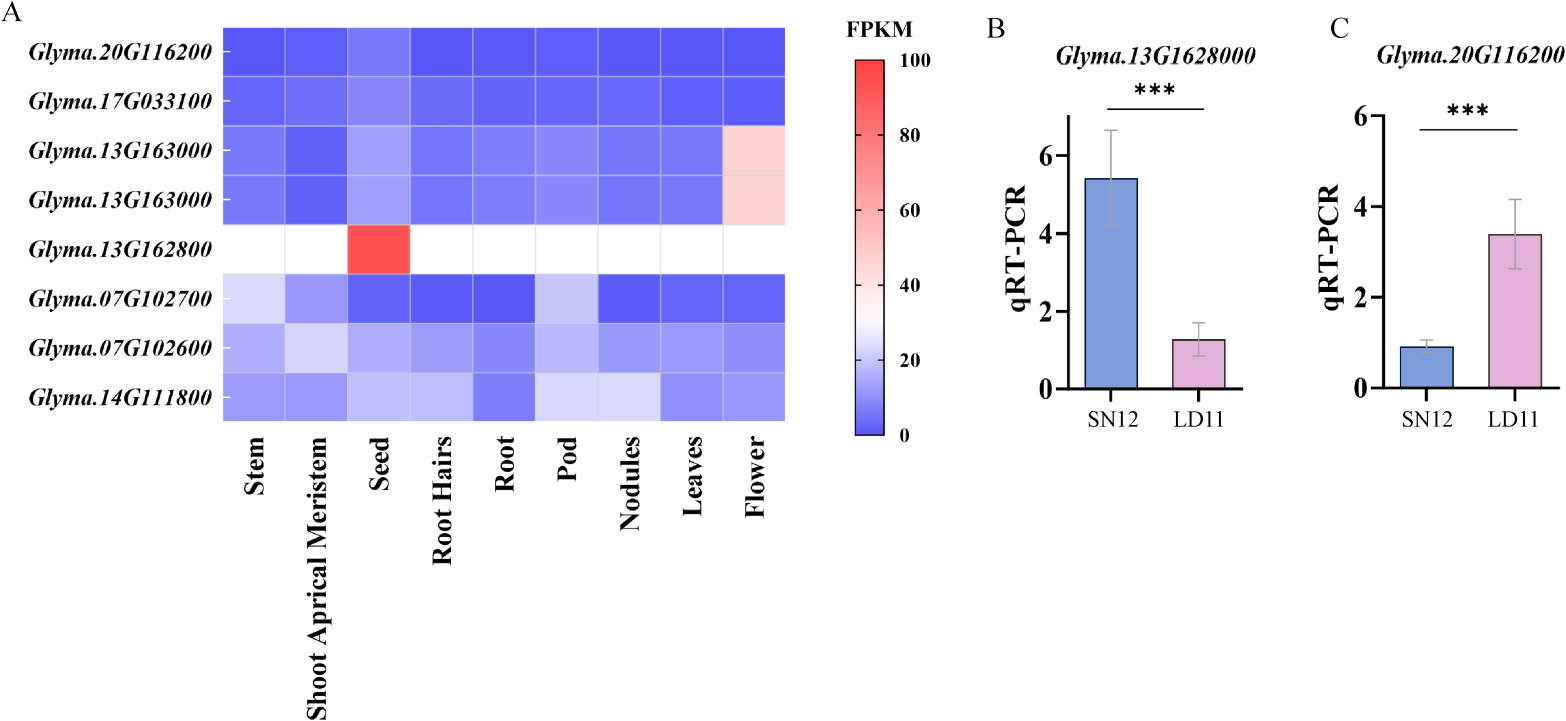
**(A)** Heatmap of candidate gene expression levels in different soybean tissues. **(B, C)** The qRT−PCR analysis of the *Glyma.13G1628000* and *Glyma.20G116200*. ***(p < 0.001)

Using publicly accessible expression datasets from Phytozome (https://phytozome-next.jgi.doe.gov/), the transcriptional patterns of these candidate genes in different soybean tissues were obtained. *Glyma.13G1628000* showed elevated and tissue–specific expression during the soybean reproductive growth stage, particularly in seeds. In contrast, *Glyma.20G116200* exhibited high expression exclusively in seeds (**Figure 6A**). These findings suggested specific roles of *Glyma.13G1628000* and *Glyma.20G116200* in soybean seed development and yield formation. To confirm the reliability of the selected candidate genes, quantitative real–time (qRT–PCR) was utilized to examine alterations in the expression levels of *Glyma.13G1628000* and *Glyma.20G116200* in grains at R6 stage across different varieties. In Liaodou 11 (LD 11, a variety with larger HSW but fewer seeds per plant) and Shennong 12 (SN 12, a variety with smaller HSW but more seeds per plant), *Glyma.20G116200* and *Glyma.13G1628000* exhibited notable differential expression patterns among genotypes with distinct alleles (**Figures 6B, C**). Therefore, they were considered as potential genes regulating pod−related traits; however, the functions of candidate genes require further validation to confirm their roles.

### 3.6 Development of KASP markers for SNPs

**Figure 7:**
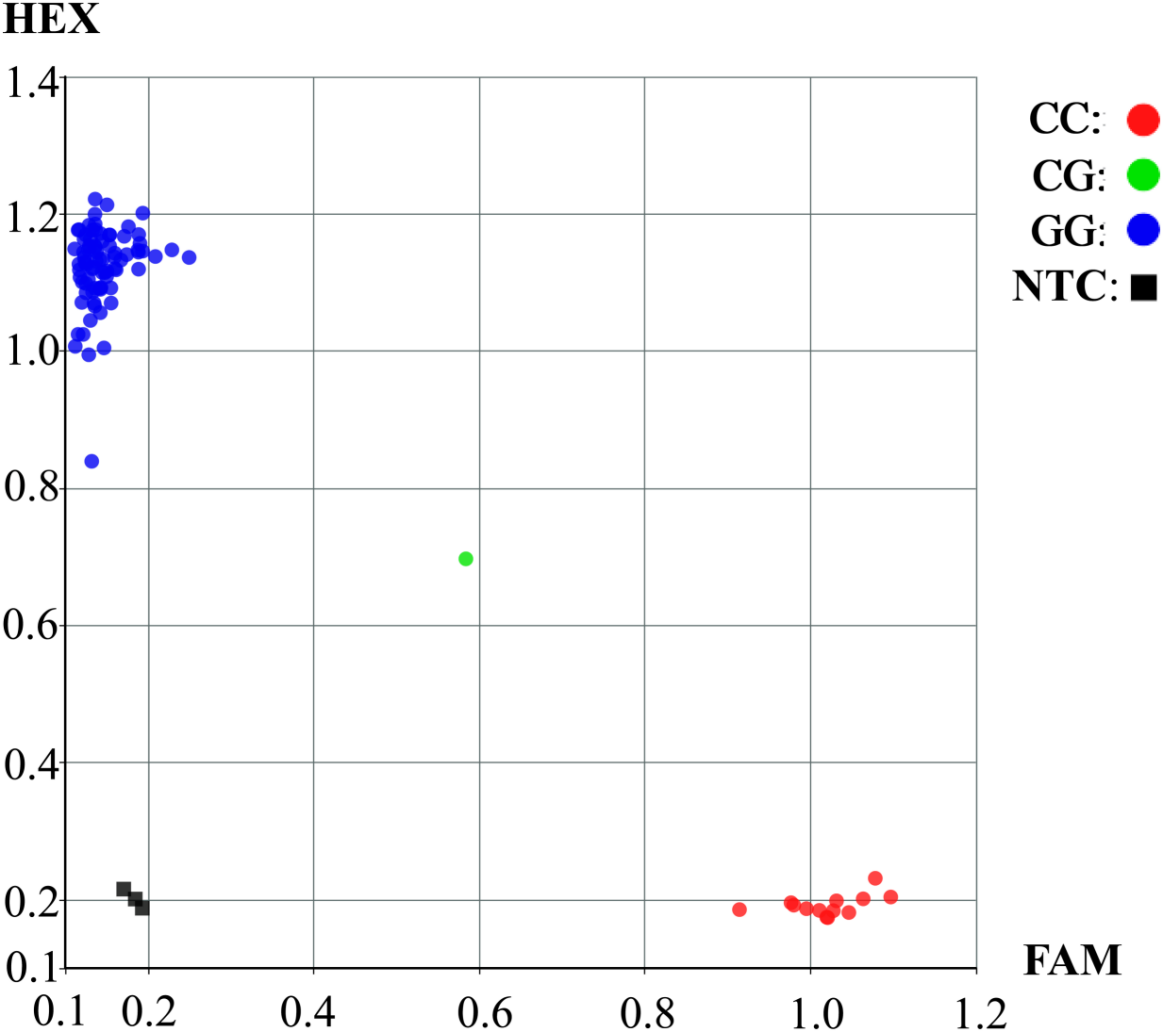
Genotyping of the KASP marker S20_35808042. NTC, negative control.

Based on above findings, a KASP marker for S20_35808042 (G/C) was developed. This locus, which is significantly associated with HSW, is located in the coding region of the *Glyma.20G116200* gene and harbors a non−synonymous mutation, allowing clear differentiation of the two genotypes at this locus. In **Figure 7**, blue dots represent soybean germplasm carrying the G allele at the mutation locus, indicating a higher HSW. In contrast, red dots represent soybean germplasm carrying the C allele at the mutation locus, exhibiting a trend toward lower HSW. Overall soybean accessions carrying the CC genotype tended to show lower HSW than those possessing the GG genotype. This finding aligned with the outcomes obtained from the haplotype analysis of the *Glyma.20G116200* locus. The clear genotype clustering pattern illustrated in the figure indicated that the designed KASP marker enabled precise SNP−based genotyping, thereby offering important insights for downstream genetic research and crop improvement programs.

## 4 Discussion

### 4.1 Phenotypic variation of soybean pods−related traits under different planting densities

This study focused on uncovering the genetic architecture underlying pod-associated traits in soybean. These characteristics are typical quantitative traits, governed by numerous genetic components and strongly modulated by environmental influences (Cao et al., 2022). The current investigation applied two planting densities (300,000 and 150,000 plants/ha) to evaluate phenotypic variation in five pod−related traits among 338 soybean accessions. Significant differences and abundant variation in these traits were observed within the population, and similar phenotypic trends were detected under both planting densities at the same location (**Table 1**). All traits exhibited continuous variation and approximately normal distributions. Correlation analysis revealed strong and highly significant positive relationships among PNPP, SNPP, PWPP and SWPP under both planting densities across all environments. Conversely, a significant negative correlation was observed between HSW and both PNPP and SNPP (**Figure 1**), aligning with results reported in multiple earlier investigations conducted in various countries (Quijano and Morandi, 2023; Wang et al., 2020).

### 4.2 GWAS of pod−related traits in soybean

The development of high-throughput sequencing technologies and dense genotyping arrays has made it feasible to routinely apply GWAS for the identification of potential candidate genes (Alqudah et al., 2022). Association mapping has been widely employed by various researchers to investigate the genetic architecture of numerous traits, including HSW, SNPP, SWPP, PNPP and PWPP (Rodrigues et al., 2016; Zhang et al., 2024; Priyanatha et al., 2022). Most genetic analyses of soybean agronomic traits have been conducted under conventional planting densities (150,000 plants/ha). The results generated under conditions of high planting density in this research may enhance our understanding of the genetic basis controlling pod-associated characteristics in soybean. After comparing the results of GWAS with previous reports, a total of 43 significant SNPs associated with 100−seed weight (HSW) were identified under both planting densities, among which 29 SNPs were located within previously reported quantitative trait loci (QTL) regions (Luo et al., 2022; Zhang et al., 2024; Cao et al., 2022; Hacisalihoglu et al., 2018; Wu et al., 2020). For example, previous researchers identified a QTL significantly associated with HSW using a RIL population derived from a high−protein and high−yielding soybean cultivar, which overlapped with the Chr14:12805909 locus detected in the present study (Whiting et al., 2020). For SNPP, 3 out of 19 significant SNPs were located within previously reported QTL regions (Qi et al., 2014; Hao et al., 2012). Among them, the SNP Chr13:27800164 identified under both high and low−density planting conditions overlapped with the previously defined Seed weight 13 region (Wang et al., 2016), suggesting that this locus may exhibit pleiotropic effects. For seed weight per plant (SWPP), 5 out of 17 significant SNPs were located within previously reported QTL regions (Kuroda et al., 2013). In addition, 2 out of 10 SNPs associated with PNPP and 1 out of 13 SNPs associated with PWPP were also found within known QTL intervals (Hacisalihoglu et al., 2018; Zhang et al., 2010; Palomeque et al., 2009) (**Supplementary Table S2**). It is noteworthy that the loci identified in our study accounted for only about one−third of the previously reported quantitative trait loci (QTLs), further confirming the complexity and polygenic nature of soybean yield−related traits. These discrepancies may have resulted from differences in genetic backgrounds, population sizes, marker densities, environmental effects, and the GWAS models employed.

### 4.3 Candidate gene analysis for pod−related traits

Among the soybean pods−related traits, only eight significant SNPs were consistently identified for HSW, SWPP, and SNPP across at least two different planting densities or environmental scenarios (**Table 2**). Based on gene annotation analysis, five candidate genes associated with HSW were detected, including *Glyma.14G111800*, *Glyma.14G112000*, *Glyma.15G219900*, and *Glyma.20G116200*. *Glyma.14G111800* encodes aspartate aminotrans ferase 5, which is involved in the C4 photosynthetic carbon assimilation cycle and subsequently affects amino acid metabolism in plants, thereby influencing seed development (Tian et al., 2022). *Glyma.14G112000* is located within a previously reported QTL region (Whiting et al., 2020). In Arabidopsis thaliana, its homolog, aberrant lateral root formation 4 (*ALF4)*, suppresses lateral root development by interacting with RBX1 and inhibiting the activity of the SCFTIR1 complex. Mutations in *alf4* exhibit phenotypes indicative of defects in the auxin (Aux/IAA) signaling response (Bagchi et al., 2018). *Glyma.15G219900* is located within the Seed weight 15−g1 region, near the marker Sat_136 (Wu et al., 2020). This gene encodes villin 4 (*VLN4*), is highly preferentially expressed in pollen. *VLN4* serves as a key regulator of F−actin filament stability and may influence seed development by modulating pollen tube growth (Yang et al., 2025). In the analysis of seed weight per plant (SWPP), *Glyma.07G102600* and *Glyma.07G102700* are identified as potential candidate genes. *Glyma.07G102600* encodes a core component of the C/D box small nucleolar ribonucleoprotein (snoRNP) complex, which is involved in rRNA processing and ribosome biogenesis. In soybean, it functions upstream of the pre−ribosome biogenesis process through the nucleolar protein NOP56 in root apices, contributing to protein synthesis (Yin and Komatsu, 2016). *Glyma.07G102700* encodes N−myristoyltransferase (NMT), an enzyme responsible for protein N−myristoylation. In Arabidopsis thaliana, the homologous gene *NMT1* plays a critical role during post−embryonic development. Deficiency in NMT1 results in abnormal cellular polarity, hindered floral development, disturbed fruit ripening, and weakened innate immune responses (Renna et al., 2013). Among the candidate genes significantly associated with seed number per plant (SNPP), *Glyma.13G16300*0 (*GUS3*) encodes β−glucuronidase. In Arabidopsis thaliana, this gene exhibits tissue-specific expression, primarily localized in zones of vigorous cell division, suggesting a potential role in modulating the cell cycle (Woo et al., 2007).

### 4.4 Haplotype analysis of candidate genes associated with HSW and SNPP

A haplotype denotes a group of linked genetic markers inherited together on the same chromosome, and it has been extensively utilized in crop studies involving species like rice, soybean, and maize (Singh et al., 2024). Based on haplotype and sequence variation analyses, *Glyma.20G116200*, which shows a significant association with 100−seed weight (HSW), is identified as a candidate gene. It encodes a key transcriptional regulator belonging to the C2H2−type zinc finger protein family, which plays an essential role in gene expression regulation. The *JAG* gene modulates organ morphology, including leaf and petal development, by enhancing marginal expansion and stimulating cellular proliferation (Jeong et al., 2012). In soybean, the genetic locus controlling leaflet shape is closely linked to the locus governing the SNPP. Jeong et al. (2011) demonstrated through linkage analysis that the JAG gene induces the proliferation of lateral organ tissues, thereby influencing leaflet morphology and seed number in soybean. In *Brassica napus*, the *JAGGED* (*JAG*) gene as a key regulator within the seed shattering control network. CRISPR/Cas9−induced knockout mutants exhibit altered pod morphology, characterized by enlarged cells, uneven surface texture, and reduced fruit length (Zaman et al., 2019). We hypothesize that natural variation in the *JAG* gene may contribute to phenotypic variation in HSW in soybean. The significant locus Chr20_35828042 resides within the exon of the candidate gene and harbors a nonsynonymous variant. Haplotype analysis identified Hap3, which is associated with a lower 100−seed weight (HSW) phenotype (**Figure 4**). In recent years, breeders have increasingly favored the development of density−tolerant soybean cultivars, which are characterized by smaller 100−seed weight (HSW). Therefore, Hap3 may represent an elite haplotype of *Glyma.20G116200*. Moreover, the qRT−PCR results reveal differential expression of this gene among accessions with contrasting 100−seed weight (HSW). *Glyma.13G163000* (*AVP1*) encodes a vacuolar H^+^− pyrophosphatase in Arabidopsis thaliana, which has been shown to enhance abiotic stress tolerance. In cotton, transgenic plants overexpressing *AVP*1 exhibited enhanced tolerance to cold and salt stress, accompanied with increased field yield (Pasapula et al., 2011). The only SNP identified in *Glyma.13G163000* results in a nonsynonymous mutation, and the Hap2 haplotype is associated with a higher SNPP, indicating that Hap2 is a favorable haplotype. This finding was further supported by qRT−PCR analysis. Single nucleotide polymorphisms (SNPs), as functional genetic variants, serve as a valuable basis for developing molecular markers used in marker−based selection aimed at improving pod-related traits in soybean. The application of superior haplotypes identified through such variants further facilitates the breeding of high−yield soybean cultivars.

### 4.5 KASP markers for HSW

In this study, a KASP marker was developed based on SNPs associated with soybean pod traits identified through GWAS. This marker has been successfully applied for genotyping (**Figure 7**), and SNP S20_35808042 is significantly associated with HSW. This marker serves as an effective tool for enhancing yield−oriented soybean breeding and facilitates greater efficiency and precision in marker-assisted selection. Nevertheless, KASP markers targeting additional pod−related traits remain undeveloped, highlighting the necessity for continued exploration and validation to identify robust markers for these traits.

## 5 Conclusions

A GWAS conducted under high and low planting densities on 338 soybean accessions identified 103 significant SNP loci for pod-related traits. A total of eight significant SNPs were repeatedly detected, which were significantly associated with SNPP, SWPP, and HSW. Meanwhile, two genes, *Glyma.20G116200* and *Glyma.13G162800*, were identified as being significantly associated with HSW and SNPP, respectively. In addition, a KASP marker, S20_35808042 (G/C), was developed and validated. These findings offer a theoretical framework for deciphering the genetic underpinnings of pod−related traits in soybean and establish a basis for advancing molecular breeding and the development of high−yielding cultivars.

## Data Availability

The variation data reported in this paper have been deposited in the Genome Variation Map (GVM) in National Genomics Data Center, Beijing Institute of Genomics, Chinese Academy of Sciences and China National Center for Bioinformation, under accession number GVM001210 (https://bigd.big.ac.cn/gvm/getProjectDetail?Project=GVM001210).
